# Athletes and adversities: athletic identity and emotional regulation in time of COVID-19

**DOI:** 10.1007/s11332-020-00677-9

**Published:** 2020-08-31

**Authors:** Sergio Costa, Giampaolo Santi, Selenia di Fronso, Cristina Montesano, Francesco Di Gruttola, Edoardo Giorgio Ciofi, Luana Morgilli, Maurizio Bertollo

**Affiliations:** 1 Department of Neuroscience, Imaging and Clinical Sciences, University “G. D’Annunzio” of Chieti-Pescara, Chieti, Italy; 2 Department of Life Quality Studies, University “Alma Mater Studiorum” of Bologna, Bologna, Italy; 3 Behavioral Imaging and Neural Dynamics (BIND) Center, Department of Medicine and Aging Sciences, University “G. D’Annunzio” of Chieti-Pescara, Via Polacchi, 11, Chieti, Italy; 4 IMT School for Advanced Studies, Lucca, Italy; 5 Independent Sport Psychology Consultant, Rome, Italy

**Keywords:** Sport psychology, Lockdown, Coping, Athlete isolation, Performance

## Abstract

**Background:**

The COVID-19 pandemic impacted on the sporting field, with athletes constrained in home isolation without the possibility to train and compete in their usual environments. This situation has been investigated within the theoretical frameworks of athletic identity and cognitive emotion regulation.

**Objectives:**

The objectives of our investigation were to: (a) validate the athletic identity measurement scale (AIMS) for use in Italian language; and (b) explore differences by gender, typology of sport (individual vs. team), and competitive level (elite vs. non-elite) in athletic identity and in cognitive emotion regulation during the Covid-19 lockdown period.

**Methods:**

To achieve these objectives, the reliability and construct validity of the Italian version of the AIMS have been tested in Study 1. Multivariate and univariate analyses were run to evaluate differences between different groups of athletes in Study 2.

**Results:**

Results from Study 1 suggest a 3-factor higher order model of athletic identity. Results from Study 2 highlight that, during this lockdown period, elite athletes and team sports athletes show higher athletic identity. Cognitive emotion regulation strategies are different for gender and for competitive level. Finally, athletes with higher athletic identity tend to ruminate and catastrophize more.

**Conclusions:**

The present multi-study paper contributes to the theoretical field with a validated measure of athletic identity in Italian language. It also provides some practical implications that could apply in this situation of isolation and can be extended to cases such as those of injury or illness.

## Introduction

The circulation of the new SARS-COV-2 virus during the last months of 2019 affected the entire world. The Corona Virus Disease (COVID-19) changed the habits of the citizens [1] in the majority of Europe and made Italy one of the most impacted countries. This pandemic situation also affected the sporting field and athletes’ lives, competitive calendars and routines, presenting challenges and issues associated with social isolation, limited and mostly denied access to effective training environments, partners and teammates [2].

After the suspension of some championships and the postponement for a year of the European Football Championship 2020 and the Olympic and Paralympic Games Tokyo 2020, several Italian teams and federations allowed their athletes to return home, where they conducted mandatory home isolation following government guidelines. Several researchers have investigated the impact that such inactivity conditions may have on physiological systems, as well as on athletic performance [3]. However, how this worrisome situation might affect also athletes’ emotional regulation and their Athletic Identity is still to be explored, not knowing when it will be safe to return to athletic participation after the lockdown situation is overcome. “Moreover, after investigating perceived stress and psychobiosocial states scholars suggested to explore specific emotion regulation and coping strategies of athletes in this time of adversities [4].”

Based on the mentioned considerations, the aim of the current study was to examine Italian athletes’ identity and their emotional experience during the COVID-19 crisis. For this purpose, we refer to the psychological constructs of Athletic Identity [5, 6] and Cognitive Emotion Regulation [7, 8]. The construct of athletic identity is rooted in the wider literature on self-concept and is defined as the degree to which a person identifies with the athlete role [5]. The Athletic identity measurement scale (AIMS [6]) is one of the most prominent instruments used to assess Athletic Identity. It has been developed and refined over the last three decades, measuring three dimensions labelled “social identity”, “exclusivity” and “negative affectivity” [5, 6, 9]. This scale has been in widespread use in the sport domain and has been recently validated across several countries [10–13], however an Italian translation of this instrument has yet to be validated. For these reasons, we decided to adopt the AIMS scale for the present research, and we tested its psychometric properties in a first study.

On the other hand, Cognitive Emotion Regulation is intended as a set of cognitive strategies people use to cope with negative life events [7]. These strategies are measured in the cognitive emotion regulation questionnaire (CERQ [8]) through nine conceptually distinct subscales. This scale has already been used within the sport domain [14], and Balzarotti and colleagues validated a version in Italian language [15]. Therefore, in a second study, we focused on differences by gender, sport (i.e., individual vs. team sports) and competitive level (i.e., elite vs. non-elite athletes) in Athletic Identity and cognitive emotion regulation, within a large sample of Italian athletes. Moreover, we explored if participants differing in Athletic Identity (i.e., lower scorers vs. higher scorers) reported different cognitive emotion regulation strategies.

Athletic Identity is considered a relatively stable personality trait and develops through the years [16, 17]. However, Brewer and colleagues [18] reported some intercollegiate athletes to divest of their athletic identity, after incurring in a poor sporting season. This might be the case of the present season; the COVID-19 lockdown period lasted, in Italy, for more than 2 months and might have affected athletes’ goals and achievements. Additionally, past literature explored Athletic Identity together with coping strategies [19] and emotional aspects [20] but no links were found in artistic gymnasts or recreational exercisers, respectively.

By exploring these aspects in this particular period, we expected to observe stronger Athletic Identity in those athletes competing at higher levels, as this already emerged in the research on the topic [21]. Although it would be novel in the specific literature of Athletic Identity, we can also hypothesize that athletes in team sports may experience more negative affectivity in this period of isolation, due to the fact that they miss the social aspects of their sports. This could also impact team sports athletes’ emotional coping [22]. Differences by gender and by competitive level in the CERQ have also been found in past studies and might emerge during this lockdown period: women, despite being less severely affected by COVID-19 and with a lower mortality rate [23], may adopt less adaptive emotion regulation strategies [15] whereas athletes competing at higher levels may have more adaptive emotional coping [14]. The link between athletic identity and cognitive emotion regulation has yet to be explored and it can provide helpful implications for both the research literature and professional practice in the field.

## Study 1

### Methods

#### Sample

A sample of 392 Italian athletes (*n* = 207 women, *n* = 185 men) was surveyed for the present study immediately after the beginning of the Italian lockdown (9th of March). Participants were from 18 to 50 years old (mean age = 27.41; SD = 8.34) and came from different individual (e.g., golf, tennis, swimming and others) and team sports (e.g., basketball, rugby, soccer and others) and competitive levels (local, regional, national, international). According to the typology of sport, participants were divided into individual sports athletes (*n* = 190) and team sports athletes (*n* = 202). According to their competitive level, participants were classified as elite athletes (*n* = 210), which included national and international athletes, and non-elite athletes (*n* = 182), including athletes competing at local and regional levels. This categorization was based on the athletes’ highest standard of performance suggested by Swann and colleagues’ suggestions [24]. Within 4 weeks, some of these participants (*n* = 182) were surveyed a second time for test–retest reliability analyses.

#### Measures and procedure

The participants were asked to complete a demographic information form and the AIMS adapted for use within an Italian speaking population. Participants were also asked to provide a personal code to help researchers link the data of the first and the second data collection for the test–retest analyses. The AIMS [6] is a self-report measure designed to assess both the strength and exclusivity of identification with the athlete role. It is composed of seven items, divided in three subscales: “social identity” (items one, two and three), “exclusivity” (items four, five) and “negative affectivity” (items six, seven), with a seven-point Likert scale answer format, ranging from 1 (completely disagree) to 7 (completely agree). This scale produces both scores for the sub-dimensions and aggregated score of Athletic Identity. The translation of the AIMS was conducted using the forward–backward translation method [25] by two Italian English-speaking researchers and a native English speaker with a good command of Italian. The original version of the scales was translated independently by the researchers and then the translated text was discussed extensively. When a consensus on a pre-version of the questionnaire was reached, it was reverse translated by a native English speaker. The original English scale, the translated and retranslated texts were examined carefully for accuracy. These were discussed until agreement on the changes was reached. The final version of the questionnaire is reported in Table 1. Participants were recruited by phone, email or social network using our informal and professional networks. They received a brief description of the study together with an informed consent module. After providing consent, participants completed an online questionnaire. By means of this approach, we were able to reach a wide sample of athletes of different ages, sports and competitive levels. Participants were also asked to provide their consent for being contacted via email for a follow-up, and 182 of them took part in a second assessment for examining the reliability of the AIMS over time. In this follow-up, participants were asked to complete again the AIMS, along with demographic information, and provide again the personal code to help researchers to associate their answers with the first data collection.

**Table 1:** Original and Italian versions of the AIMS, with means, standard deviations, Cronbach alphas and test–retest correlations

Athletic identity measurement scale				
Original item	Italian item	Mean (SD)	Cronbach alpha	Test–retest correlation
Social identity				
1. I consider myself an athlete	1. Mi considero un atleta	5.71 (1.38)	0.64	0.71
2. I have many goals related to my sport	2. Mi pongo molti obiettivi rispetto allo sport che pratico	5.77 (1.31)		0.74
3. Most of my friends are athletes	3. La maggior parte dei miei amici sono atleti	4.59 (1.52)		0.82
Exclusivity				
4. Sport is the most important part of my life	4. Lo sport è la parte più importante della mia vita	5.18 (1.42)	0.87	0.79
5. I spend more time thinking about sport than anything else	5. Passo più tempo a pensare allo sport che ad ogni altra cosa	4.59 (1.54)		0.77
Negative affectivity				
6. I feel bad about myself when I do poorly in sport	6. Mi sento male se non mi esprimo al meglio nello sport	5.49 (1.43)	0.75	0.79
7. I would be very depressed if I were injured and could not compete in sport	7. Sarei molto depresso se fossi infortunato e non potessi competere nello sport	5.60 (1.59)		0.81

#### Data analysis

Due to the online completion procedure, which made compulsory to answer to any item, no missing values were identified in the data sample. However, some cases were removed because they reported to be amateur athletes. Data were then analysed using IBM SPSS 20.0 for observing distribution and reliability. Further data analysis was performed using IBM AMOS Graphic 20.0 to examine the factor structure of the scale through confirmatory factor analysis (CFA). CFA tests provide a fit for the model, and, in particular, it is considered excellent to achieve: a ratio lower than 3 between Chi-square and degrees of freedom; Comparative Fit Index (CFI), Normed Fit Index (NFI) and Tucker-Lewis Index (TLI) values equal or greater than 0.95; A Root Mean Square Error of Approximation (RMSEA) value lower than 0.05 with upper- and lower-bound confidence interval (CI) grouped tightly around the RMSEA and with a pclose value equal or above 0.5 [26].

### Results

Examination of histograms, and values of skewness and kurtosis showed that further parametric tests were allowed. The scale demonstrated good reliability in terms of internal consistency for the aggregate score (Athletic Identity, α = 0.82) and acceptable to good reliability (Cronbach alpha) for the independent scores (“social identity” = 0.64; “exclusivity” = 0.87; “negative affectivity” = 0.75). Test–retest reliability analysis also showed acceptable to good Pearson’s correlations, ranging from 0.71 to 0.82 (see full results in Table 1).

**Fig. 1:**
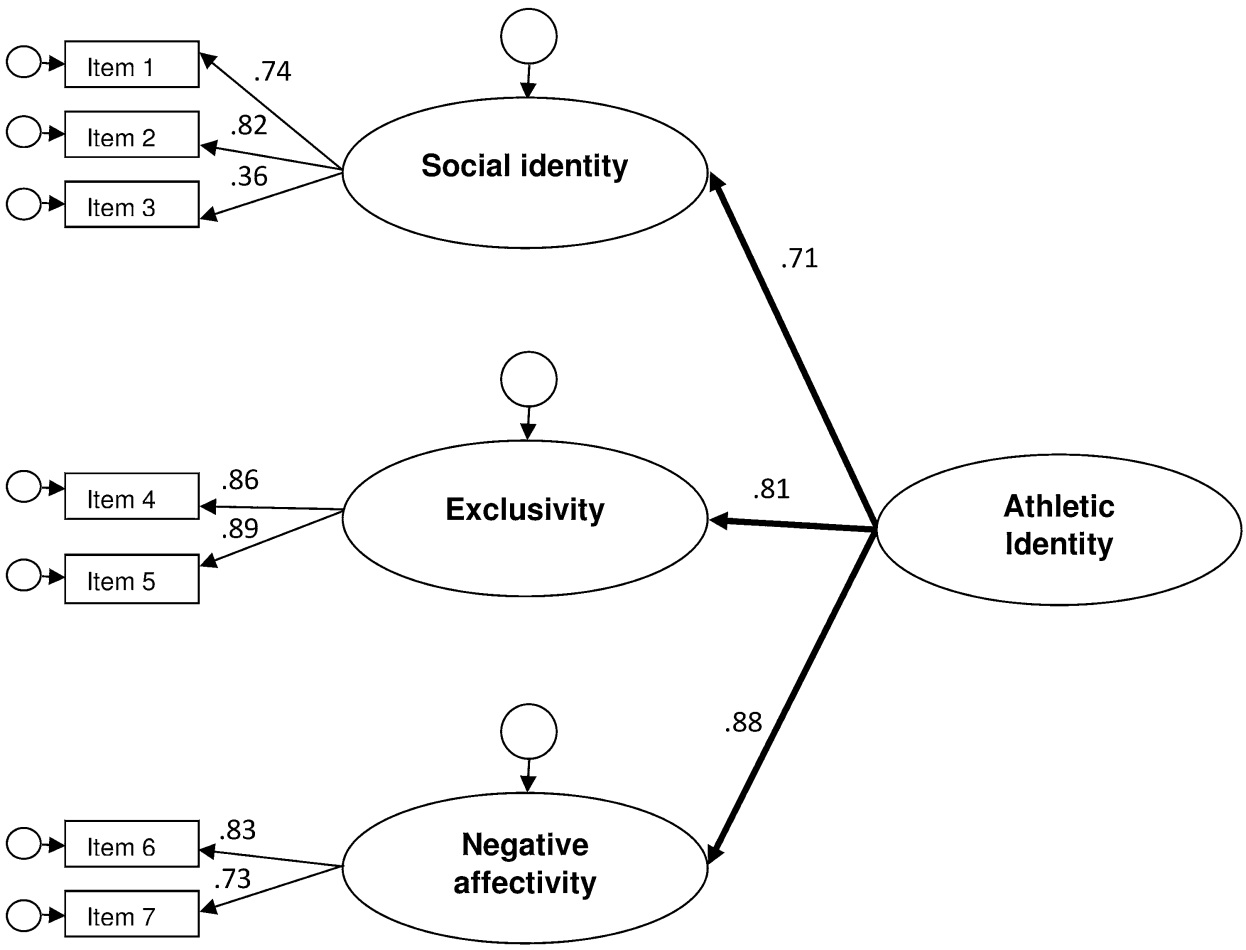
Higher order structural model of the Italian translation of the Athletic Identity Measurement Scale. Note. Standardized estimates are reported in the figure. Model fit: *χ*^2^ = 20.5(11), *χ*^2^/df = 1,87, *p* < 0.05; CFI = 0.99; NFI = 0.98; TLI = 0.98; RMSEA = 0.047 [90% CI = 0.011–0.078], pclose = 0.52

Confirmatory Factor Analyses (CFAs) run with Amos Graphic evidenced an excellent fit for the model proposed by Brewer and Cornelius [6] with Athletic Identity as a higher order factor and “social identity”, “exclusivity”, and “negative affectivity” as sub-dimensions [Model fit: *χ*^2^ = 20.5(11), *χ*^2^/df = 1.87, *p* < 0.05; CFI = 0.99; NFI = 0.98; TLI = 0.98; RMSEA = 0.047 (90% CI = 0.011–0.078), pclose = 0.52]. Factor loadings for each item were significant and standardized regression weights ranged between 0.36 and 0.82 for the “social identity” sub-dimension, between 0.86 and 0.88 for “exclusivity”, and between 0.73 and 0.83 for “negative affectivity”. Standardized regression weights for the general construct of Athletic Identity on the three sub-dimensions were also significant and ranged from 0.71 to 0.88 (see Fig. 1 for full details). The same excellent fit emerged for a lower order model with “social identity”, “exclusivity”, and “negative affectivity” as correlated dimensions, highlighting the possibility to adopt either an aggregate score for the Athletic Identity construct or independent scores for the subscales.

### Discussion

Overall, these results confirmed the reliability and the construct validity of the AIMS also for use within an Italian speaking population of athletes. The reliability of the instrument is supported both by an overall acceptable reliability in term of internal consistency and a good stability in terms of test–retest reliability after a follow-up within 4 weeks. The factor structure which emerged is in line with that suggested by Brewer and Cornelius [6], and the fact that the ‘Goodness of Fit’ for the Italian model is largely above the fit for the original model (Model fit reported by the authors: *χ*^2^ = 40.0(13), *χ*^2^/df = 3.08; CFI = 0.94; NFI = 0.91; TLI = 0.90; RMSEA = 0.11), highlights the quality of the translation and data collection performed for the present study. Further research should explore other properties of the Italian version of the AIMS (i.e., convergent, discriminant, predictive and nomological validities) to strengthen the validity of this instrument.

The three-factor higher order structure is also in line with what emerged in recent cross-cultural validations [10, 11, 13] and consolidates the construct of Athletic Identity and the validity of the AIMS across different countries and cultures. It should be noted that in recent years, some authors have suggested an expansion of this instrument [12], with the addition of a further sub-dimension (i.e., “positive affectivity”) and a split of “social identity” into “social” and “self-identity”. However, these studies need to be extended and the three-factor model proposed by Brewer and Cornelius [6] remains the more consolidated to date.

## Study 2

### Methods

#### Sample

One thousand one hundred twenty-five athletes were surveyed for Study 2 (*n* = 610 women, *n* = 515 men). The age of participants ranged from 18 to 50 years (mean age = 27.47; SD = 8.47) and athletes were participating in various sports (e.g., baseball/softball, basketball, beach volleyball, boxing, climbing, cycling, dancesport, fencing, field hockey, futsal, golf, horse riding, martial arts, rhythmic and artistic gymnastic, rowing, rugby, running, shooting gallery, skating, soccer, swimming, tennis, track and field, triathlon, volleyball, water polo, weightlifting and different competitive levels (local, regional, national, international). In line with Study 1 and with previous literature on the topic [23], athletes were divided according to the typology of sport (*n* = 539 individual sports athletes, *n* = 586 team sports athletes) and their competitive level (*n* = 572 elite athletes—national, international; *n* = 553 non-elite athletes—regional, local).

#### Measures and procedure

When they were still under the Covid-19 lockdown period (late April and early May), participants completed a battery of online questionnaires, comprising a socio-demographic questionnaire, the Italian version of the AIMS (see Study 1), and an Italian version of the Cognitive Emotion Regulation Questionnaire (CERQ [15]). The socio-demographic questionnaire collected information on the following variables: gender, date of birth, type of sport and level. The Italian version of the AIMS is fully described in Study 1. The Italian version of the CERQ is a 27-item self-report measure designed to assess individual differences in cognitive regulation of emotions in response to stressful, threatening or traumatic life events. The instrument assesses nine three-item dimensions: “positive reappraisal”, “putting into perspective”, “positive refocusing”, “planning”, “acceptance”, blaming others”, “self-blame”, “rumination”, and “catastrophizing”. The items are rated on a Likert scale ranging from 1 (almost never) to 5 (almost always). Participants were recruited using the snowball sampling technique [27], in which the researchers start with a known group of people, using their own informal and professional networks, and these subjects recruit subsequent participants among their acquaintances. In addition, we used social networks and a collaboration with the School of Sport of the Italian Olympic Committee (Scuola dello Sport-Comitato Olimpico Nazionale Italiano, SDS-CONI) for recruiting people online. The challenges relating to online surveys are the sampling, response rate, non-respondent characteristics, maintenance of confidentiality, and ethical issues [28]. To avoid incomplete or no responses, the answers to all items were made compulsory on a Google form. Finally, data were stored and carefully manually cleaned to remove unwanted cases (e.g., amateur athletes and repeated cases) prior to data analysis. The study was conducted in accordance with the Declaration of Helsinki and received approval by the institutional review board of our research centre.

#### Data analysis

Data were analysed using IBM SPSS 20.0 for exploring distribution and reliability of the scales. Differences based on gender, typology of sport (individual vs. team), and competitive level (elite vs non-elite) were then explored through Multivariate analysis of variance (MANOVA). In a first analysis, a 2 × 2 × 2 matrix (gender X typology of sport X competitive level) was adopted for observing differences in “social identity”, “exclusivity” and “negative affectivity” (scales of the AIMS) as dependent variables. In a second MANOVA, a 2 × 2 × 2 matrix was run with gender, typology of sport, and competitive level as fixed factors and the nine dimensions of the CERQ as dependent variables. Finally, athletes were divided according to the median value on the Athletic Identity total score, into those with ‘higher Athletic Identity’ and those with ‘lower Athletic Identity. The choice to divide lower scorers and higher scorers based on the median value is in line with Field’s suggestions for slightly negative distributed data [29]. A one-way ANOVA was performed to observe differences between higher and lower scorers on the ratings in the CERQ dimensions.

### Results

Sampling distribution was examined through an overall observation of histograms, skewness and kurtosis. Although data showed a slightly negative distribution, it was suitable for parametric tests [29]. Cronbach alphas for the AIMS were 0.66 for “social identity”, 0.85 for “exclusivity” and 0.74 for “negative affectivity”, with an alpha of 0.82 for the aggregated score of Athletic Identity. Cronbach alpha values for the CERQ ranged from 0.62 to 0.87 (“positive reappraisal” = 0.75, “putting into perspective” = 0.79, “positive refocusing” = 0.84, “planning” = 0.80, “acceptance” = 0.62, “blaming others” = 0.87, “self-blame” = 0.74, “rumination” = 0.70, “catastrophizing” = 0.76).

Multivariate results from the first MANOVA on the AIMS sub-dimensions evidenced significant differences for the typology of sport [Wilks’ *λ* = 0.951, *F*(3, 1115) = 19.17, *p* < 0.00, _p_*η*^2^ = 0.049, observed power > 0.999], for the competitive level [Wilks’ *λ* = 0.879, *F*(3, 1115) = 51.25, *p* < 0.00, _p_*η*^2^ = 0.121, observed power > 0.999], and for the interaction typology of sport and competitive level [Wilks’ *λ* = 0.971, *F*(3, 1115) = 11.12, *p* < 0.00, _p_*η*^2^ = 0.029, observed power = 0.999]. Univariate results showed significant differences for the typology of sport on “social identity”, “exclusivity” and “negative affectivity”, with team sports athletes scoring higher on all three sub-dimensions. Moreover, elite athletes scored higher on all AIMS sub-dimensions compared to their non-elite counterparts. The interaction ‘typology of sport X competitive level’ also produced significant differences: elite athletes reported similar “social identity” and “negative affectivity” in both individual and team sports. At the non-elite level, team sports athletes reported higher “social identity” and “negative affectivity” than individual sports athletes. No significant differences by gender were found. Full details of significant differences, including *F*, significance, _p_*η*^2^ and power, are shown in Table 2.

**Table 2:** Univariate results from MANOVA for ‘gender X typology of sport X competitive level’ on ‘social identity, ‘exclusivity’, and ‘negative affectivity’

Independent variable	Dependent variable	df	*F*	*p*	_p_*η*^2^	Observed power
Typology of sport	Social identity	(1, 1124)	20.18	0.000	0.018	0.994
	Exclusivity	(1, 1124)	6.31	0.012	0.006	0.709
	Negative affectivity	(1, 1124)	47.57	0.000	0.041	> 0.999
Competitive level	Social identity	(1, 1124)	134.55	0.000	0.108	> 0.999
	Exclusivity	(1, 1124)	84.99	0.000	0.071	> 0.999
	Negative affectivity	(1, 1124)	39.52	0.000	0.034	> 0.999
Typology of sport X Competitive level	Social identity	(1, 1124)	29.47	0.000	0.026	> 0.999
	Negative affectivity	(1, 1124)	9.25	0.002	0.008	0.860

Note only significant results are reported in the table

Multivariate results for the second MANOVA on the nine dimensions of the CERQ showed significant differences for gender [Wilks’ *λ* = 0.942, *F*(9, 1109) = 7.63, *p* < 0.00, _p_*η*^2^ = 0.058, observed power > 0.999], for the competitive level [Wilks’ λ = 0.977, *F*(9, 1109) = 2.87, *p* < 0.01, _p_*η*^2^ = 0.023, observed power = 0.967], for the interaction between gender and typology of sport (Wilks’ *λ* = 0.984, *F*(9, 1109) = 1.99, *p* < 0.05, _p_*η*^2^ = 0.016, observed power = 0.858), and for the interaction gender and competitive level [Wilks’ *λ* = 0.977, *F*(9, 1109) = 2.92, *p* < 0.01, _p_*η*^2^ = 0.023, observed power = 0.969]. Differences by gender emerged on the CERQ dimensions, with women scoring higher on “putting things into perspective” and “rumination”, and men scoring higher on “planning” and “blaming others”. Differences by competitive level were also found, with elite athletes reporting more “planning”, “acceptance”, and non-elite athletes reporting more “self-blame”. The interaction “gender X typology of sport” showed that women scored higher on “catastrophizing” in individual sports, whereas men reported more “catastrophizing” in team sports. Finally, the interaction “gender X competitive level” evidenced differences in: “acceptance” with elite men scoring higher than non-elite men, and women showing similar results; and in “catastrophizing”, with non-elite women scoring higher than elites. No significant differences were found based on the typology of sport, on the interaction “typology of sport and competitive level”, or on the interaction “gender, typology of sport and competitive level”. Full details of significant univariate results for this analysis, including *F*, significance, _p_*η*^2^ and power are shown in Table 3.

**Table 3:** Univariate results from MANOVA for ‘gender X typology of sport X competitive level’ on the dimensions of the cognitive emotion regulation questionnaire

Independent variable	Dependent variable	df	*F*	*p*	_p_*η*^2^	Observed power
Gender	Putting into perspective	(1, 1124)	4.98	0.026	0.004	0.607
	Planning	(1, 1124)	8.86	0.003	0.008	0.845
	Blaming others	(1, 1124)	9.73	0.002	0.009	0.876
	Rumination	(1, 1124)	19.28	0.000	0.017	0.992
Competitive level	Planning	(1, 1124)	12.46	0.000	0.011	0.941
	Acceptance	(1, 1124)	8.08	0.005	0.007	0.811
	Self-blame	(1, 1124)	6.11	0.014	0.005	0.695
Gender X Typology of sport	Catastrophizing	(1, 1124)	10.69	0.001	0.009	0.904
Gender X Competitive level	Acceptance	(1, 1124)	4.15	0.042	0.004	0.531
	Catastrophizing	(1, 1124)	10.51	0.001	0.009	0.900

Note only significant results are reported in the table

**Fig. 2:**
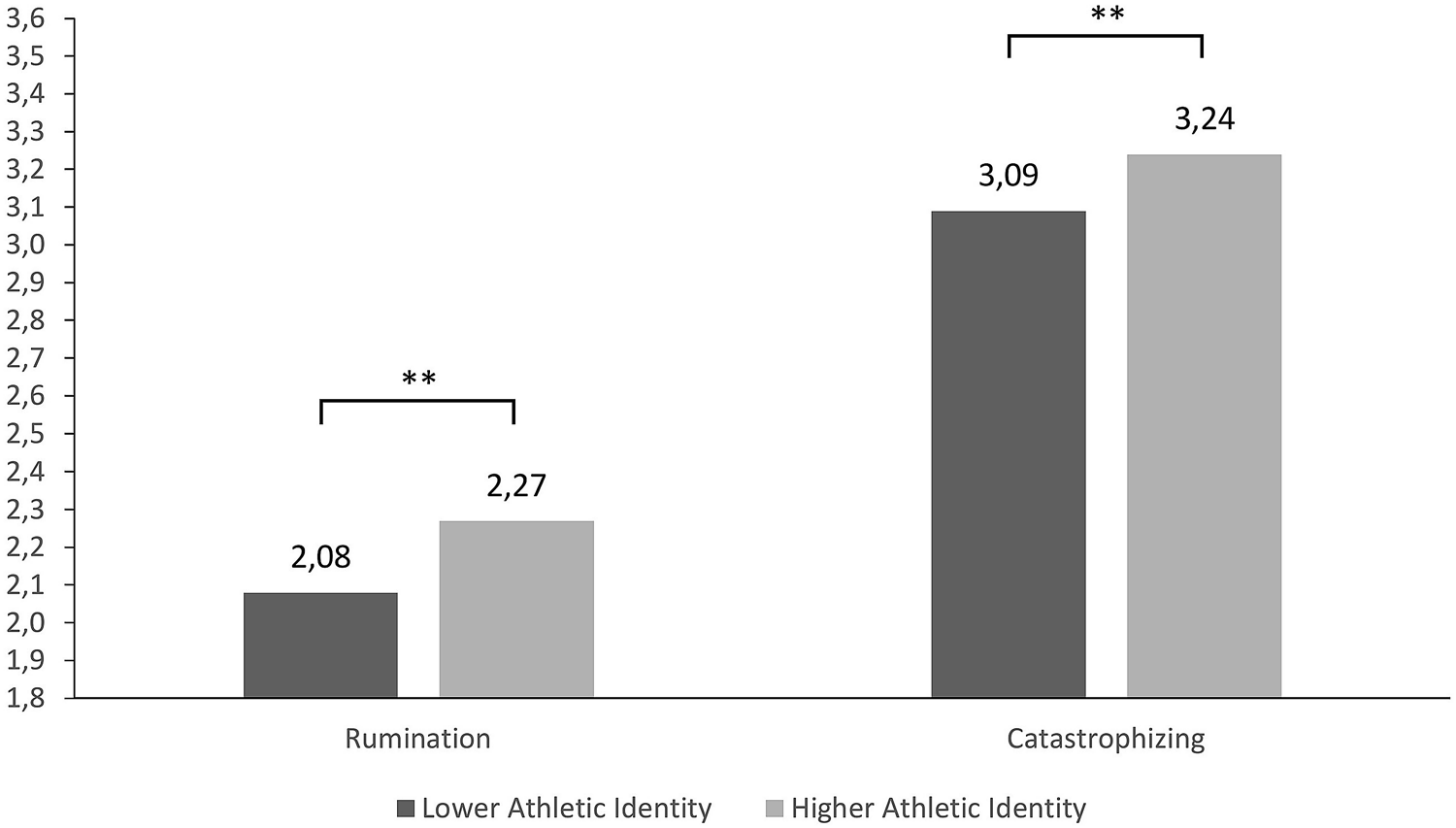
Differences in “rumination” and “catastrophizing” between athletes with higher Athletic Identity and athletes with lower Athletic Identity. Note. *p < 0.05, **p < 0.01, ***p < 0.001

The last analysis aimed at exploring the differences between those athletes higher in Athletic Identity total scores and those scoring lower on the scale. A one-way ANOVA was run to observe differences in the reports of Cognitive Emotion Regulation and significant difference were found for the “rumination” dimension, *F*(1, 1124) = 8.50, *p* < 0.01, _p_*η*^2^ = 0.008, observed power = 0.830 and for the “catastrophizing” dimension, *F*(1, 1124) = 12.66, *p* < 0.00, _p_*η*^2^ = 0.011, observed power = 0.945. In both cases, athletes with higher Athletic Identity tended to ruminate and catastrophize more than athletes with lower Athletic Identity (see Fig. 2). For all other dimensions of the CERQ, no significant differences were found.

### Discussion

During the Covid-19 lockdown, elite athletes showed significantly higher levels of “social identity”, “exclusivity” and “negative affectivity” sub-dimensions of the AIMS, compared to non-elite athletes. Past literature on the topic [21] evidenced recreational athletes experiencing the same “negative affectivity” as elite athletes. The fact that, in our research, elite athletes also scored higher on “negative affectivity” may be due to the peculiarities of this period of isolation. The absence of significant gender differences on Athletic Identity is also in line with the literature, although a previous study evidenced higher identity in male student-athletes than in their female colleagues [30]. Moreover, team sports athletes scoring higher than individual sports athletes on all the sub-dimensions of the AIMS appears to be something novel in the literature of the topic. Over this period of isolation, we could expect to find higher “negative affectivity” in team sports athletes because of the denied access to their teammates. However, team sports athletes also strengthened their “social identity” and “exclusivity”, probably this was compensating for the social and physical distance from their teams.

With regards to Cognitive Emotion Regulation strategies, gender differences had already been found in Balzarotti and colleagues’ research [15], with women reporting to use more “rumination” and “catastrophizing”. These two results are somewhat in line with the present study, in which women athletes were more prone to ruminate and, in individual sports, to catastrophize. These gender differences in Cognitive Emotion Regulation strategies may be due to the fact that women tend to express their emotions more than men [31], and this period of social isolation might have been an obstacle to this expressivity. In addition, in our survey, women athletes emerged also as being more able to put things into perspective, whereas men used more the cognitive strategy of planning (i.e., to think about what steps to take and how to handle the negative event). The differences by typology of sport and competitive level on the CERQ that emerged in this research are novel in the literature, as the CERQ has not yet reached widespread use in the sporting field. The fact that elite athletes scored higher in “planning” and “acceptance”, and lower in “self-blame”, is in line with Shirvani and colleagues [14], who reported a more adaptive Cognitive Emotion Regulation in semi-professional athletes when compared to amateur athletes. In the literature, elite athletes have emerged as having better strategies to emotionally cope with stressful situations [32]. This may have reflected in more adaptive Cognitive Emotion Regulation strategies over the COVID-19 lockdown period.

Finally, the finding that athletes with higher Athletic Identity tend to ruminate and catastrophize more can be considered novel in the literature. This may be due to the fact that, in this period of isolation, athletes feel more affected by being far from their usual training and competitive environments. This evidence can be relevant to identify those athletes who are more at risk and may benefit for psychological support. It also provides practical implications to address sport psychologists in their interventions: it may be indicated to reduce the athletes’ investment in their athletic role to avoid maladaptive cognitive emotion regulation strategies, such as catastrophizing and rumination.

## Conclusions

The present studies contribute both to the theoretical and applied field of sport psychology. First, they provide researchers and practitioners with a reliable measurement instrument that is valid for the assessment of Athletic Identity among an Italian speaking population. The excellent fit that emerged for the Italian version of the AIMS extends further the cross-cultural validity of this instrument, giving new possibilities for sport specific research in the Italian context. Second, findings highlight some practical implications to be considered when working with athletes during this period of isolation or in similar periods, such as illness or injury, in which athletes experience a variety of psychological features [33]. For example, the fact that athletes with higher Athletic Identity tend to ruminate and catastrophize more during this particular period, could suggest helping them to reduce their identification with their athletic role. This is in line with Brewer and colleagues [34], who highlighted that some athletes reduced their investment in the athletic role to protect their self-image following anterior cruciate ligament reconstruction. Although there are some analogies with injury situations, the period of COVID-19 lockdown has its peculiarities. For injured athletes, for example, it is important the support normally received from family, friends, teammates, and peers [33]. However, this social support could have been lacking over this period of isolation. In addition, a variety of other factors could have had an influence, such as the fact that the lockdown was mandatory and the social isolation was not due to the athletes’ physical impossibility.

Some limitations should be noted. For example, it is acknowledged that a more balanced sense of identity is a protective factor over disruptive life changes [16]; however, in the present research, only Athletic Identity has been considered in relation to Cognitive Emotional Regulation. Taking into consideration and measuring other aspects of self-concept, such as identities relating to culture, ethnicity, race, gender, or religion, could have provided us with a wider understanding of this issue. To explore these aspects of the self, it would have been necessary to adopt other existing scales validated for an Italian speaking population, but there appears to be a lack of these measures with a sole religious identity measure [35] recently validated. On the other hand, it would have been possible to validate more general measures of self-concept for the Italian context, such as the Tennessee Self-concept scale (TSCS-2 [36]), but we considered a priority for the Italian sporting context the translation of the AIMS, which is a scale specific for the sport domain and has received widespread use across several countries [10–13].

Future studies should explore beyond the limitations of the present paper and investigate how different aspects of the self (e.g., self-efficacy) interact with Athletic Identity and relate to athletes’ well-being and behaviours. In fact, since its inception in the sport psychology literature, Athletic Identity has been debated as a “Hercules’ muscle” or, vice versa, as an “Achille’s heel” [5] with studies highlighting stronger Athletic Identity either to lead to higher commitment to sport and greater athletic performances [37] or to impede the development of a multi-dimensional self-concept and pose at greater risk of emotional distress [37, 38]. Other studies linked a high and exclusive Athletic Identity to difficulties in retiring from sport [17]. On the other hand, recent studies have suggested that athletes might struggle to adapt in other areas of their lives if they engage solely with sport goals, but readjusting their goals may help them to alleviate distress associated with life changes [39]. To conclude, it will be important that next studies provide practical recommendations to athletes to reduce the unwanted consequences of the forced quarantine and how to organize a service of support for their mental health [40]. It would be relevant also considering the opportunity for online intervention strategies, which have been already explored in the sporting literature as a way to help emotion regulation [41] and may be a resource in those cases where athletes are constrained at home.

## Abbreviations

AIMS: Athletic identity measurement scale
CERQ: Cognitive emotion regulation questionnaire
